# Homœopathic Impertinence

**Published:** 1850-11

**Authors:** 


### ARTICLE VIII.

### HOMŒOPATHIC IMPERTINENCE.

The following correspondence appears in one of the Homœopathic Journals with copious comments. It speaks for itself:

St. Charles, Ill., Sept. 12, 1850.

Dr. N. S. Davis—Sir: I am a Homœopathist, from a conviction of the *truth* of the *principle*, and the *efficacy* of the *practice* of Homœopathia. With these views, will you graduate me if I comply with the ordinary requisitions of the Faculty?

Yours, &c., M. DANIEL COE.

Chicago, Sept. 16th, 1850.

M. Daniel Coe—Dear Sir: I am directed to inform you that the Faculty of Rush Medical College *will not* recommend you to the Trustees for a Degree, so long as they have any reason to suppose that you entertain the doctrines, and intend to trifle with human life on the principles you avow in your letter. To do otherwise would involve both parties in the grossest inconsistency. Very respectfully yours,

N. S. DAVIS,
Secretary of the Faculty of Rush Med. College.

